# Effects of exercise interventions on fall-related injuries in community-dwelling older adults: an updated systematic review and meta-analysis

**DOI:** 10.3389/fpubh.2026.1916495

**Published:** 2026-08-26

**Authors:** Beihai Ge, Jinsong Luo, Xiaofeng Cheng, Deng Wang, Renming Liu, Xianhui Liao

**Affiliations:** 1 Rugao People's Hospital Affiliated to Kangda College of Nanjing Medical University, Nantong, Jiangsu, China; 2 Wuchang University of Technology, Wuhan, Hubei, China; 3 Department of Infectious Diseases, Shangyu People's Hospital of Shaoxing, Shaoxing University, Shaoxing, China; 4 School of Physical Education, Guangzhou University of Sport, Guangzhou, China; 5 Neuromodulation Research Unit, Department of Physiotherapy, School of Primary and Allied Health Care, Monash University, Melbourne, VIC, Australia; 6 Department of Physiotherapy, School of Primary and Allied Health Care, Monash University, Melbourne, VIC, Australia

**Keywords:** exercise, falls, fractures, injury prevention, meta-analysis, older adults

## Abstract

**Background:**

Exercise interventions are widely recommended for preventing falls in older adults in the community; however, existing evidence regarding their effectiveness in reducing fall-related injuries remains inconsistent. This updated systematic review and meta-analysis aimed to assess the impact of exercise interventions on fall-related injuries in older adults in the community.

**Methods:**

PubMed, Embase, Cochrane Library, and CINAHL databases were systematically searched from inception to April 2, 2026. Included studies were randomized controlled trials of exercise interventions for older adults in the community. Outcome measures included all fall-related injuries, injuries requiring medical attention, severe injuries, and fall-related fractures. Pooled risk ratios (RRs) and 95% confidence intervals (CIs) were calculated using a random-effects model. Risk of bias was assessed using the Cochrane RoB 2 tool, and the quality of evidence was assessed using the GRADE method. This review followed the PRISMA 2020 guidelines and was registered in PROSPERO (CRD420261348337).

**Results:**

29 randomized controlled trials involving 6367 participants were included. Exercise intervention significantly reduced the incidence of all fall-related injuries based on 19 studies [RR = 0.87, 95% CI (0.80, 0.95), I^2^ = 15.7%], injuries requiring medical attention based on 11 studies [RR = 0.71, 95% CI (0.57, 0.89), I^2^ = 33.3%], severe injuries based on 8 studies [RR = 0.67, 95% CI (0.54, 0.83), I^2^ = 0.0%], and fall-related fractures based on 11 studies [RR = 0.56, 95% CI (0.37, 0.85), 23.3%]. Heterogeneity among outcome measures was low to moderate.

**Conclusion:**

Exercise intervention can effectively reduce the incidence of fall-related injuries and fractures in older adults in the community and may contribute to healthy aging and injury prevention. Keywords: exercise; falls; fractures; older adults; injury prevention; meta-analysis.

**Systematic review registration:**

PROSPERO, identifier [CRD420261348337].

## Introduction

Falls are one of the most common and serious public health problems affecting older adults worldwide, and a leading cause of disability and death among this population (1). Approximately one-third of older adults in the community fall at least once a year, and the incidence of falls increases significantly with age (2). Fall-related injuries, including fractures, head injuries, and injuries requiring medical attention, are leading causes of functional decline, loss of independence, hospitalization, and death in older adults (3). In addition to serious physical consequences, falls are often accompanied by significant psychological impacts, such as fear of falling, reduced physical activity, and decreased quality of life, all of which can have long-term effects on mental and physical health (4). With the rapid growth of the global aging population, falls and fall-related injuries place a significant economic burden on healthcare systems and have become a major challenge for healthy aging and public health worldwide (5).

Exercise interventions are widely recognized as one of the most effective non-pharmacological strategies for preventing falls in older adults (6, 7). Previous randomized controlled trials, systematic reviews, and meta-analyses have shown that exercise programs, particularly those incorporating balance training, functional training, resistance training, and multi-component interventions, can significantly reduce the incidence and risk of falls among older adults in the community (8, 9). Therefore, exercise-based fall prevention strategies have been incorporated into numerous international clinical practice guidelines and public health recommendations for healthy aging, and are widely used in community fall prevention programs (10).

However, a reduction in the number of falls does not necessarily translate to a corresponding reduction in fall-related injuries (11). Fall-related injuries—especially serious injuries and fractures—have greater clinical significance than falls alone because they are directly associated with healthcare resource utilization, long-term disability, residency in aged care facilities, and mortality (3, 12). From both clinical and public health perspectives, preventing fall-related injuries may be more important than simply reducing the number of falls (13). Although a systematic review published in 2013 suggested that exercise interventions may reduce the risk of fall-related injuries among older adults in the community, the review included 17 randomized controlled trials and separately evaluated all fall-related injuries, injuries requiring medical care, serious injuries, and fall-related fractures. Although beneficial effects were observed across these injury outcomes, the evidence was based on a relatively limited number of studies (14).

Since the publication of that review, additional randomized controlled trials investigating the effects of different exercise modalities on fall-related injuries have been published, substantially expanding the available evidence base. Furthermore, advances in systematic review methodology, including the PRISMA 2020 statement, the Cochrane RoB 2 risk of bias assessment tool, and the GRADE framework for evaluating the certainty of evidence, provide opportunities for a more comprehensive and rigorous reassessment of the current evidence. Over the past decade, numerous randomized controlled trials have evaluated the effects of various exercise modalities (including home exercise, multi-component exercise, balance training, and other functional exercise interventions) on fall-related injuries, increasing the available evidence since the previous review. In addition, the literature search has been updated to April 2, 2026, and the number of included randomized controlled trials has increased from 17 to 29. Furthermore, this review applies the Cochrane RoB 2 tool to assess the risk of bias and the GRADE approach to evaluate the certainty of evidence. Compared with the previous review, injury outcomes were also categorized into all fall-related injuries, injuries requiring medical attention, severe injuries, and fall-related fractures to provide a more comprehensive evaluation of the effects of exercise interventions (7, 10). Meanwhile, significant advancements have been made in the methodological standards for systematic reviews and meta-analyses, including the widespread adoption of the PRISMA 2020 statement, the RoB 2 risk of bias assessment tool, and the GRADE framework for assessing the certainty of evidence (15–17). These developments underscore the necessity for an updated and methodologically rigorous synthesis of existing evidence.

Therefore, we conducted an updated systematic review and meta-analysis to comprehensively assess the impact of exercise interventions on fall-related injuries in older adults in the community (18, 19). Specifically, we examined the effects of exercise interventions on all types of injuries, falls requiring medical attention, severe injuries, and fall-related fractures, an updated and comprehensive synthesis of the current evidence updated and high-quality evidence for fall injury prevention strategies in older adults (20, 21).

## Methods

### Program registration and reporting guidelines

The design and reporting of this systematic review and meta-analysis followed the Preferred Reporting Items for Systematic Reviews and Meta-analyses (PRISMA 2020) (15) statement. The study protocol was registered with the International Registry for Prospective Systematic Reviews (22) (PROSPERO; Registry No: CRD420261348337) on March 22, 2026, prior to the commencement of the literature search. No deviations from the registered protocol were made during the conduct of this review.

### Search strategy

We conducted a systematic literature search in PubMed, Embase, the Cochrane Library, and CINAHL databases, covering the period from database inception to April 2, 2026. The search strategy combined Medical Subject Headings (MeSH) with free-text keywords related to falls, exercise interventions, injuries, and older adults. Key search terms included combinations of “falls,” “exercise,” “physical activity,” “training,” “Tai Chi,” “injury,” “fracture,” and “older adults.” The search strategy was tailored to each database. Where applicable, randomized controlled trials (RCTs) were used to improve search specificity. Detailed search strategies for all databases are available in the supplementary materials, and corresponding search strategies for other databases were developed accordingly. In addition, the reference lists of all included studies and relevant systematic reviews were manually screened to identify potentially eligible studies that might have been missed during the electronic database searches. No language or publication year restrictions were applied. Gray literature and trial registries were not searched. The complete search strategies for all databases are provided in Supplementary Table 1.

### Inclusion criteria

Inclusion criteria were developed according to the PICOS framework (population, intervention, control group, outcome measure, and study design).

Studies meeting the following criteria were included: Population: Community-dwelling older adults aged 60 years and older, the study meets the inclusion criteria if the average age of the participants is ≥60 years. Intervention: Structured exercise interventions designed to prevent falls among community-dwelling older adults, including balance training, resistance training, Tai Chi, walking programmes, and other structured exercise programmes, were eligible. General physical activity advice or education alone was not considered an eligible intervention. Studies evaluating multicomponent interventions in which the independent effect of exercise could not be isolated were excluded. Control Group: Routine care, no intervention, health education, or a non-movement control intervention. Outcomes: Fall-related injuries, including:(1) All falls leading to injury; (2) Falls requiring medical attention; (3) Falls leading to serious injury; (4) Fall-related fractures. Study Design: Randomized controlled trials (RCTs).

### Exclusion criteria

Studies were excluded if they met the following criteria: Included participants residing in nursing homes or long-term care facilities; Focused primarily on patients with specific neurological disorders (e.g., stroke/cerebrovascular accident, Parkinson's disease, Alzheimer's disease, or dementia) or other conditions that could substantially affect mobility, balance, or fall risk; Were non-randomized studies, conference abstracts, reviews, study protocols, or meta-analyses; Did not provide extractable outcome data relevant to this review are shown in Supplementary Table 2.

### Data management, screening and selection, and extraction

All retrieved records were managed using EndNote, Covidence, and Microsoft Excel throughout the review process (23). Duplicate records were identified and removed prior to screening. The overall study selection process was documented using a PRISMA 2020 flow diagram (15).

Two reviewers (JL and XC) independently screened titles and abstracts according to the predefined eligibility criteria. Full-text articles considered potentially eligible were subsequently assessed independently by the same reviewers. Any disagreements during the screening and study selection process were resolved through discussion. When consensus could not be reached, a third reviewer (BG) made the final decision.

Data extraction was independently performed by two reviewers (JL and XC) using a standardized data extraction form developed for this review. The data extraction form was pilot-tested using a sample of eligible studies before formal data extraction to ensure consistency and completeness of data collection. Although no multiple publications reporting data from the same study population were identified among the included studies, we predefined that, if such publications had been identified, they would have been assessed together and treated as a single study. The publication containing the most complete outcome data would have been used as the primary source, with additional reports consulted to supplement missing information where appropriate. When multiple injury-related outcomes were reported, outcomes were classified according to the framework used in previous reviews as follows: All fall injuries; Falls requiring medical attention; Severe fall injuries; Fall-related fractures. When necessary, the corresponding authors of the included studies were contacted to obtain missing or ambiguous data.

The following information was extracted from each included study: first author, publication year, country, sample size, participant characteristics (including mean age or age range), intervention characteristics (type, frequency, duration, and intensity where available), comparator characteristics, follow-up duration, and outcome measures related to fall-related injuries.

Reasons for exclusion at the full-text review stage were recorded and documented in the PRISMA flow diagram.

### Risk of bias assessment

Two reviewers (Deng Wang and Renming Liu) independently assessed the methodological quality of the included randomized controlled trials using the Cochrane Risk of Bias Assessment Tool 2 (RoB 2) (16). Each included study was independently evaluated based on a detailed review of the full-text article. When available, the study protocol and trial registration records were also consulted to support the risk-of-bias assessment. The domains assessed included: Bias due to the randomization process; Bias due to deviations from the expected intervention; Bias due to missing outcome data; Bias in outcome measurement; Bias in the selection of reported outcomes. The risk of bias for each study was rated as “low risk,” “some issues,” or “high risk.” according to the RoB 2 guidance. Any disagreements were resolved through discussion.

### Statistical analysis

All statistical analyses were performed using Stata software (version 18.0; StataCorp LLC, College Station, TX, USA). Risk ratios (RRs) with corresponding 95% confidence intervals (CIs) were used as the summary measure of effect because all included outcomes were dichotomous and were consistently reported as the number of participants who sustained at least one fall-related injury. The analyses were therefore based on the number of participants with injuries, rather than the total number of injury events. When recurrent injuries occurred, the data reported in the original studies were used without additional adjustment. A DerSimonian–Laird random-effects model was applied to pool effect estimates to account for potential clinical and methodological heterogeneity among studies. Statistical heterogeneity was assessed using Cochran's Q test and quantified using the I^2^ statistic. Heterogeneity was interpreted as low (I^2^ < 25%), moderate (25%−50%), substantial (50%−75%), or considerable (>75%). All included studies reported risk ratios (RRs); therefore, no hazard ratios (HRs) or rate ratios were included, and no conversion of effect measures was required.

No studies with double-zero events or multi-arm designs were identified among the included trials. When studies reported outcomes at multiple follow-up time points, data from the final follow-up assessment were extracted for quantitative synthesis.

Sensitivity analyses were conducted using a leave-one-out approach, in which each study was sequentially omitted to evaluate the influence of individual studies on the pooled effect estimates and to assess the robustness of the results. Publication bias was evaluated by visual inspection of funnel plots when at least 10 studies were available for an outcome. A two-sided *P* value < 0.05 was considered statistically significant.

### Definition of evidence

The determination of evidence for each outcome was independently assessed by Jinsong Luo and Xiaofeng Cheng assessed using the Graded Assessment, Development, and Evaluation of Recommendations (GRADE) methodology (17). Based on risk of bias, inconsistency, indirectness, imprecision, and publication bias, the certainty of evidence was rated as high, moderate, low, or very low. Any disagreements between the two reviewers were resolved through discussion until consensus was reached.

## Results

### Study screening

**Figure 1:**
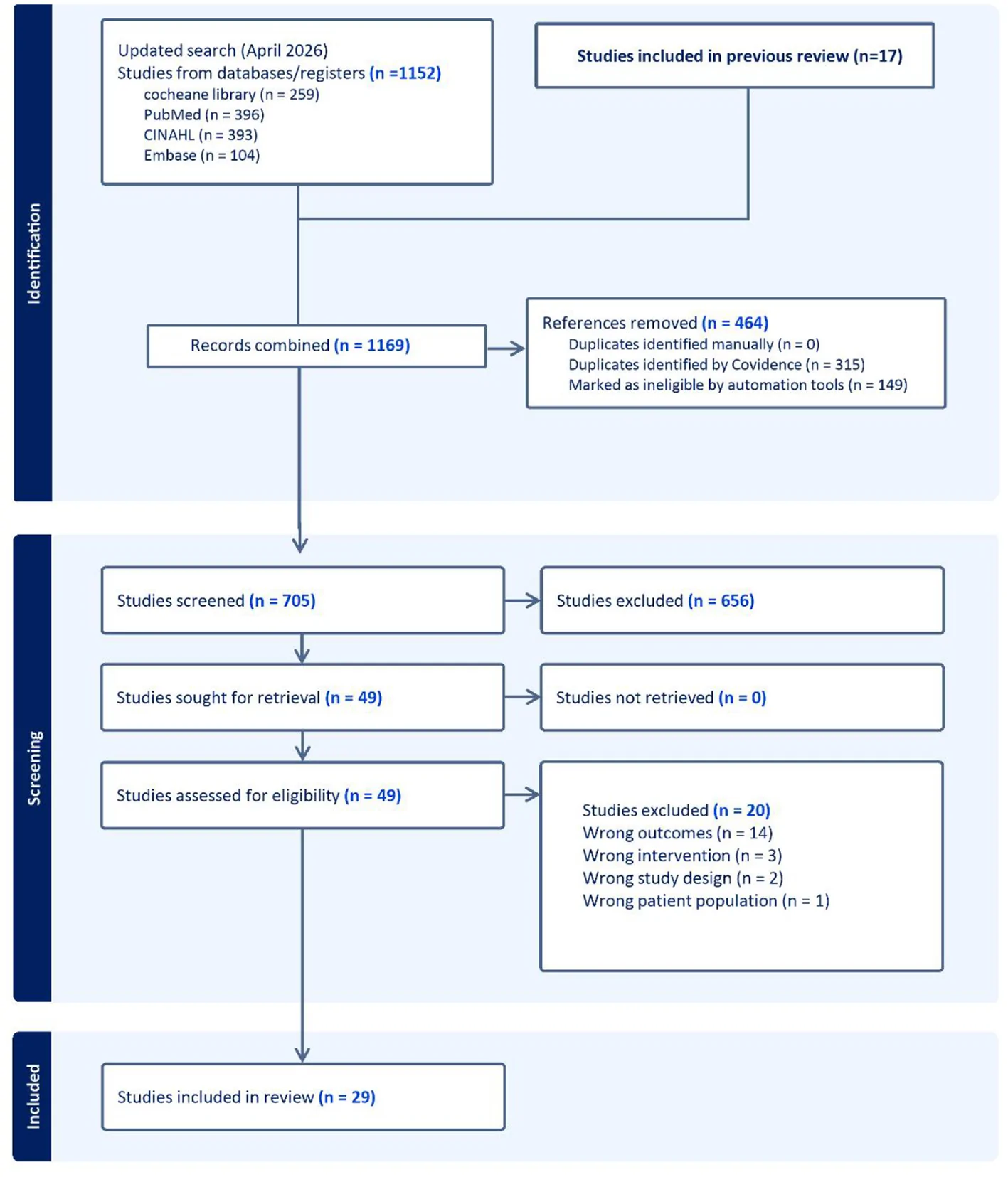
Flowchart of literature screening and inclusion for systematic review and meta-analysis (15).

Database searches retrieved 1152 records entries from PubMed (*n* = 396), Embase (*n* = 104), Cochrane Library (*n* = 259), and CINAHL (*n* = 393). In addition to the updated database search, 17 studies included in the previous systematic review were considered for eligibility. After combining records from the updated search and previous review, a total of 1,169 records were identified. Among these, 464 duplicate records were removed, including 315 duplicates identified by Covidence and 149 records marked as ineligible by automation tools, leaving 705 records for title and abstract screening. After title and abstract screening, 656 records were excluded, and 49 full-text articles were retrieved and assessed for eligibility. No full-text articles were unavailable. Among the 49 full-text articles assessed, 20 studies were excluded due to wrong outcomes (*n* = 14), wrong intervention (*n* = 3), wrong study design (*n* = 2), and wrong patient population (*n* = 1). Finally, 29 reports representing 29 unique randomized controlled trials were included in the systematic review and meta-analysis. The study screening process is shown in the PRISMA 2020 flowchart (Figure 1).

Compared to the previous systematic review published in 2013 (14), this update included more randomized controlled trials published over the past decade, thus expanding the evidence base for exercise interventions for fall-related injuries among older adults in the community.

### Study characteristics

The 29 included randomized controlled trials involved a total of 6,367 participants. These studies were conducted in multiple countries and evaluated various exercise interventions, including balance training, strength training, multi-component exercise programs, Tai Chi, and home-based exercise interventions. The duration of interventions and follow-up periods varied across studies. The included trials were conducted across 14 countries. Australia and Finland each contributed 17.2% of included studies, followed by the United States (13.8%) and the United Kingdom (10.3%). A summary of the overall characteristics of the included randomized controlled trials, including the number of studies, sample size, countries, and intervention categories, is presented in Table 1.

**Table 1:** Characteristics (40–68).

Characteristics	Number (%)	Mean
Total sample size	6,366 (100)	
Age, years		75.02
Sex		
Male participants, *n* (%)	1,009 (15.84)^*^	
Female participants, *n* (%)	4,808 (75.51)^*^	
Country		
Australia	5 (17.2)	
Finland	5 (17.2)	
United States	4 (13.8)	
Britain	3 (10.3)	
Germany	2 (8.70)	
New Zealand	2 (8.70)	
France	1 (3.4)	
Netherlands	1 (3.4)	
China	1 (3.4)	
Hungary	1 (3.4)	
Thailand	1 (3.4)	
Canada	1 (3.4)	
Denmark	1 (3.4)	
Japan	1 (3.4)	
Language		
English	29 (100)	

^*^: Fitzharris et al. (44); Suttanon et al. (68) data on age and sex not reported. General participant and study characteristics (*n* = 29).

Most studies included older adults aged 65 and over residing in the community, with some trials having most female participants. Control groups typically received routine care, health education, social visits, or low-intensity activities not specifically designed for fall prevention in Tables 2, 3.

**Table 2:** Characteristics of the included studies.

References (Language, Location)	Sample characteristics			
	Sample size	Sex	Age	Intervention/Control
Barnett et al. (40) (English, Australia)	I/C: 83/80	I (Male/female): 25/58; C (Male/female): 29/51	I: 74.4 ± 4.9 C: 75.4 ± 6.0	I: Group exercise C: usual care
Campbell et al. (41) (English, New Zealand)	I/C: 117/116	I (Male/female): 0/117; C (Male/female): 0/116	I: 84.1 ± 3.4 C: 84.1 ± 3.1	I: Home exercise C: usual care
Cornillon et al. (42) (English, France)	I/C: 116/150	I (Male/female): 26/124; C (Male/female): 26/127	I: 71.3 ± 4.4 C: 70.9 ± 4.4	I: Group exercise C: usual care
Fitzharris et al. (44) (English, Australian)	I/C: 135/137	I (Male/female): NR; C (Male/female): NR	I: NR C: NR	I: Group exercise C: usual care
Freiberger et al. (45) (English, Germany)	I/C: 63/80	I (Male/female): 33/30; C (Male/female): 43/37	I: 76.1 ± 4.1 C: 76.8 ± 4.1	I: Home exercise C: usual care
Haines et al. (48) (English, Australia)	I/C: 19/34	I (Male/female): 14/5; C (Male/female): 18/16	I: 80.9 ± 8.9 C: 80.5 ± 6.5	I: Group exercise C: usual care
Kemmler et al. (50) (English, Germany)	I/C: 123/123	I (Male/female): 0/123; C (Male/female): 0/123	I: 68.9 ± 3.9 C: 69.2 ± 4.1	I: Home exercise C: usual care
Korpelainen et al. (52) (English, Finland)	I/C: 83/76	I (Male/female): 0/83; C (Male/female): 0/76	I: 72.9 ± 1.1 C: 72.8 ± 1.2	I: Group exercise C: usual care
Li et al. (53) (English, United States)	I/C: 125/131	I (Male/female): 38/87; C (Male/female): 39/92	I: 76.94 ± 4.69 C: 77.99 ± 5.14	I: Group exercise C: usual care
Luukinen et al. (57) (English, Finland)	I/C: 243/243	I (Male/female): 52/191; C (Male/female): 49/194	I: 88 ± 3 C: 88 ± 3	I: Group exercise C: usual care
McRae et al. (43) (English, United States)	I/C: 24/38	I (Male/female): 0/42; C (Male/female): 0/38	I: 72.4 ± 0.9 C: 70.0 ± 0.9	I: Group exercise C: usual care
McMurdo et al. (58) (English, Britain)	I/C: 44/48	I (Male/female): 0/44; C (Male/female): 0/48	I: NR C: NR	I: Group exercise C: usual care
Means et al. (59) (English, United States)	I/C: 181/157	I (Male/female): 78/103; C (Male/female): 68/89	I: 73.5 C: 73.5	I: Group exercise C: usual care
Robertso et al. (65) (English, New Zealand)	I/C: 119/121	I (Male/female): 39/80; C (Male/female): 39/82	I: 81.1 ± 4.5 C: 80.8 ± 3.8	I: Home exercise C: usual care
Skelton et al. (66) (English, Britain)	I/C: 50/31	I (Male/female): 0/50; C (Male/female): 0/31	I: 72.7 ± 5.8 C: 73.2 ± 5.4	I: Group exercise C: usual care
Smulders et al. (67) (English, Netherlands)	I/C: 50/46	I (Male/female): 45/5; C (Male/female): 45/1	I: 70.5 ± 5.0 C: 71.6 ± 4.4	I: Group exercise C: usual care
Wolf et al. (56) (English, Australia)	I/C: 100/97	I (Male/female): 0/100; C (Male/female): 0/97	I: 71.6 ± 5.5 C: 71.7 ± 5.3	I: Group exercise C: usual care
Gregori et al. (47) (English, United States)	I/C: 60/60	I (Male/female): 31/29; C (Male/female): 32/28	I: 71.3 ± 4.7 C: 71.5 ± 4.6	I: Group exercise C: usual care
Karinkanta et al. (49) (English, Finland)	I/C: 37/35	I (Male/female): 0/37; C (Male/female): 0/35	I: 73.6 C: 72.7	I: Group exercise C: usual care
Patil et al. (62) (English, Finland)	I/C: 103/102	I (Male/female): 0/103; C (Male/female): 0/102	I: 74.8 ± 2.9 C: 73.8 ± 3.1	I: Group exercise C: usual care
Li et al. (54) (English, Chian)	I/C: 35/35	I (Male/female): 10/25; C (Male/female): 11/24	I: 77.89 ± 10.38 C: 80.57 ± 8.93	I: Group exercise C: usual care
Mikó et al. (60) (English, Hungary)	I/C: 50/50	I (Male/female): 0/50; C (Male/female): 0/50	I: 69.33 ± 4.56 C: 69.10 ± 5.3	I: Group exercise C: usual care
Rikkonen et al. (64) (English, Finland)	I/C: 457/467	I (Male/female): 0/457; C (Male/female): 0/467	I: 76.4 ± 3.3 C: 76.6 ± 3.2	I: Group exercise C: usual care
Suttanon et al. (68) (English, Thailand)	I/C: 131/146	I (Male/female): NR; C (Male/female): NR	I: 72.2 ± 5.4 C: 72.9 ± 5.6	I: Home exercise C: usual care
Liu-Ambrose et al. (55) (English, Canada)	I/C: 172/172	I (Male/female): 62/110; C (Male/female): 53/119	I: 81.2 ± 6.1 C: 81.9 ± 6.0	I: Group exercise C: usual care
Nørgaard et al. (61) (English, Denmark)	I/C: 70/70	I (Male/female): 29/41; C (Male/female): 32/38	I: 72.7 ± 4.7 C: 72.0 ± 4.7	I: Group exercise C: usual care
Patil et al. (63) (English, Britain)	I/C: 205/204	I (Male/female): 0/205; C (Male/female): 0/204	I: 74.4 ± 2.9 C: 74.0 ± 3.1	I: Group exercise C: usual care
Gianoudis et al. (46) (English, Australia)	I/C: 81/81	I (Male/female): 21/60; C (Male/female): 22/59	I: 67.7 ± 6.5 C: 67.2 ± 5.5	I: Group exercise C: usual care
Kim et al. (51) (English, Japan)	I/C: 52/53	I (Male/female): 0/52; C (Male/female): 0/53	I: 77.83 ± 4.21 C: 77.83 ± 4.15	I: Group exercise C: usual care

I, Intervention group; C, Control group; NR, (Not report).

**Table 3:** Characteristics of exercise interventions included in the studies.

References (Language, Location)	Dosage			
	Intervention/ Control	Duration (weeks)	Frequency (sessions per week)	Time (min/session or min/d)
Barnett et al. (40) (English, Australia)	I: Group exercise C: usual care	I: 48 C: NR	I: 3 C: NR	I: 60 C: NR
Campbell et al. (41) (English, New Zealand)	I: Home exercise C: usual care	I: 48 C: NR	I: 3 C: NR	I: 30 C: NR
Cornillon et al. (42) (English, France)	I: Group exercise C: usual care	I: 6 C: NR	I: 3 C: NR	I: 90 C: NR
Fitzharris et al. (44) (English, Australian)	I: Group exercise C: usual care	I: 15 C: NR	I: 1 C: NR	I: 60 C: NR
Freiberger et al. (45) (English, Germany)	I: Home exercise C: usual care	I: 16 C: NR	I: 2 C: NR	I: 60 C: NR
Haines et al. (48) (English, Australia)	I: Group exercise C: usual care	I: 18 C: NR	I: 5 C: NR	I: 60 C: NR
Kemmler et al. (50) (English, Germany)	I: Home exercise C: usual care	I: 72 C: NR	I: 2 C: NR	I: 60 C: NR
Korpelainen et al. (52) (English, Finland)	I: Group exercise C: usual care	I: 72 C: NR	I: 1 C: NR	I: 60 C: NR
Li et al. (53) (English, United States)	I: Group exercise C: usual care	I: 26 C: NR	I: 3 C: NR	I: 60 C: NR
Luukinen et al. (57) (English, Finland)	I: Group exercise C: usual care	I: 64 C: NR	I: NR C: NR	I: NR C: NR
McRae et al. (43) (English, United States)	I: Group exercise C: usual care	I: 30 C: NR	I: 3 C: NR	I: 45 C: NR
McMurdo et al. (58) (English, Britain)	I: Group exercise C: usual care	I: 6 C: NR	I: 3 C: NR	I: 90 C: NR
Means et al. (59) (English, United States)	I: Group exercise C: usual care	I: 6 C: NR	I: 3 C: NR	I: 90 C: NR
Robertso et al. (65) (English, New Zealand)	I: Home exercise C: usual care	I: 48 C: NR	I: 3 C: NR	I: 30 C: NR
Skelton et al. (66) (English, Britain)	I: Group exercise C: usual care	I: 36 C: NR	I: 2 C: NR	I: 30 C: NR
Smulders et al. (67) (English, Netherlands)	I: Group exercise C: usual care	I: 5.5 C: NR	I: 11 C: NR	I: NR C: NR
Wolf et al. (56) (English, Australia)	I: Group exercise C: usual care	I: 15 C: NR	I: 1 C: NR	I: 45 C: NR
Gregori et al. (47) (English, United States)	I: Group exercise C: usual care	I: 12 C: NR	I: 3 C: NR	I: 90 C: NR
Karinkanta et al. (49) (English, Finland)	I: Group exercise C: usual care	I: 12 C: NR	I: 3 C: NR	I: NR C: NR
Patil et al. (62) (English, Finland)	I: Group exercise C: usual care	I: 96 C: NR	I: 1 C: NR	I: 120 C: NR
Li et al. (54) (English, Chian)	I: Group exercise C: usual care	I: 24 C: NR	I: 3 C: NR	I: 40 C: NR
Mikó et al. (60) (English, Hungary)	I: Group exercise C: usual care	I: 12 C: NR	I: 3 C: NR	I: 30 C: NR
Rikkonen et al. (64) (English, Finland)	I: Group exercise C: usual care	I: 24 C: NR	I: 1 C: NR	I: 60 C: NR
Suttanon et al. (68) (English, Thailand)	I: Home exercise C: usual care	I: 12 C: NR	I: 4 C: NR	I: 60 C: NR
Liu-Ambrose et al. (55) (English, Canada)	I: Group exercise C: usual care	I: 24 C: NR	I: 3 C: NR	I: 60 C: NR
Nørgaard et al. (61) (English, Denmark)	I: Group exercise C: usual care	I: 48 C: NR	I: 4 C: NR	I: 20 C: NR
Patil et al. (63) (English, Britain)	I: Group exercise C: usual care	I: 8 C: NR	I: 3 C: NR	I: 20 C: NR
Gianoudis et al. (46) (English, Australia)	I: Group exercise C: usual care	I: 48 C: NR	I: 3 C: NR	I: NR C: NR
Kim et al. (51) (English, Japan)	I: Group exercise C: usual care	I: 12 C: NR	I: 2 C: NR	I: 60 C: NR

I, Intervention group; C, Control group; NR, (Not report).

Consistent with the framework used in the original review (14), fall-related injuries were categorized into four prespecified outcomes: (1) All injury falls, (2) Falls leading to medical attention, (3) Severe injury falls, and (4) Fall-related fractures in Table 4.

**Table 4:** Characteristics of the included studies.

References (Language, Location)	Outcome	Adverse events (Yes/No/No report)	Follow-up (Yes/No/No report)
Barnett et al. (40) (English, Australia)	All injurious falls	*n* = 13: Dropped out (*N* = 13)	follow-up: Yes
Campbell et al. (41) (English, New Zealand)	All injurious falls	*n* = 21: Dropped out (*N* = 21)	follow-up: Yes
Cornillon et al. (42) (English, France)	Falls resulting in medical care, falls resulting in serious injuries	*n* = 5: Dropped out (*N* = 5)	follow-up: Yes
Fitzharris et al. (44) (English, Australian)	All injurious falls, falls resulting in medical care	No report	No report
Freiberger et al. (45) (English, Germany)	all injurious falls	*n* = 24: Dropped out (*N* = 24)	follow-up: Yes
Haines et al. (48) (English, Australia)	All injurious falls, falls resulting in medical care, falls resulting in fractures	*n* = 3: Dropped out (*N* = 3)	follow-up: Yes
Kemmler et al. (50) (English, Germany)	All injurious falls, falls resulting in fractures	*n* = 19: Dropped out (*N* = 19)	follow-up: Yes
Korpelainen et al. (52) (English, Finland)	Falls resulting in fractures	*n* = 24: Dwithdrew consent (*N* = 10), excluded due to medical reasons (*N* = 12)	follow-up: Yes
Li et al. (53) (English, United States)	All injurious falls, falls resulting in medical care	*n* = 47: Dropped out (*N* = 47)	follow-up: Yes
Luukinen et al. (57) (English, Finland)	Falls resulting in medical care	*n* = 49: Dropped out (*N* = 49)	follow-up: Yes
McRae et al. (43) (English, United States)	Falls resulting in serious injuries	No report	No report
McMurdo et al. (58) (English, Britain)	Falls resulting in fractures	No report	No report
Means et al. (59) (English, United States)	All injurious falls	*n* = 128: Dropped out (*N* = 128)	follow-up: Yes
Robertso et al. (65) (English, New Zealand)	All injurious falls, falls resulting in medical care, falls resulting in serious injuries, falls resulting in fractures	*n* = 29: Dropped out (*N* = 29)	follow-up: Yes
Skelton et al. (66) (English, Britain)	Falls resulting in medical care	No report	No report
Smulders et al. (67) (English, Netherlands)	All injurious falls, falls resulting in serious injuries, falls resulting in fractures	*n* = 6: Dropped out (*N* = 6)	follow-up: Yes
Wolf et al. (56) (English, Australia)	Falls resulting in serious injuries	No report	No report
Gregori et al. (47) (English, United States)	All injurious falls	*n* = 11: Dropped out (*N* = 11)	follow-up: Yes
Karinkanta et al. (49) (English, Finland)	All injurious falls	*n* = 2: Dropped out (*N* = 2)	follow-up: Yes
Patil et al. (62) (English, Finland)	All injurious falls, falls resulting in fractures	No report	No report
Li et al. (54) (English, Chian)	All injurious falls	No	follow-up: Yes
Mikó et al. (60) (English, Hungary)	All injurious falls	*n* = 3: Dropped out (*N* = 3)	follow-up: Yes
Rikkonen et al. (64) (English, Finland)	All injurious falls, falls resulting in medical care, falls resulting in serious injuries, falls resulting in fractures	No report	follow-up: Yes
Suttanon et al. (68) (English, Thailand)	All injurious falls, falls resulting in medical care	No	follow-up: Yes
Liu-Ambrose et al. (55) (English, Canada)	Falls resulting in medical care, falls resulting in serious injuries	*n* = 1: Dropped out (*N* = 1)	follow-up: Yes
Nørgaard et al. (61) (English, Denmark)	All injurious falls, falls resulting in medical care, falls resulting in serious injuries, falls resulting in fractures	No	follow-up: Yes
Patil et al. (63) (English, Britain)	Falls resulting in medical care, falls resulting in fractures	*n* = 39: Dropped out (*N* = 39)	follow-up: Yes
Gianoudis et al. (46) (English, Australia)	All injurious falls	*n* = 12: Dropped out (*N* = 12)	follow-up: Yes
Kim et al. (51) (English, Japan)	Falls resulting in fractures	No report	follow-up: Yes

I, Intervention group; C, Control group; NR, (Not report).

### Risk of bias assessment

This study used the Cochrane Risk of Bias Tool 2 (RoB 2) to assess the risk of bias in five methodological domains. The risk of bias assessment was independently performed for each included randomized controlled trial by reviewing the full-text articles. When available, study protocols and trial registration records were also consulted to support the assessment. Overall, the methodological quality of the studies included was generally acceptable. Of the 29 randomized controlled trials included, 8 studies were rated as having a low overall risk of bias, 19 studies were rated as having some concerns, and 2 studies were considered to have a high risk of bias.

The domain most frequently associated with some concerns was bias due to deviations from the intended interventions, primarily because blinding of participants and intervention providers is often difficult to achieve in movement-based interventions. Some studies also showed concerns regarding missing outcome data, outcome measurement, and selection of the reported results. However, most trials demonstrated adequate randomization procedures, relatively complete outcome data, and appropriate reporting of prespecified outcomes.

**Figure 2:**
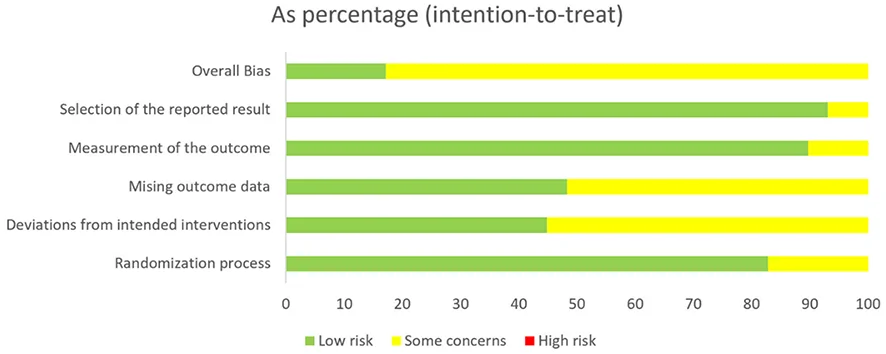
Risk of bias.

Only two studies were assessed as having a high overall risk of bias, mainly due to concerns related to deviations from intended interventions and selection of the reported results. Despite these limitations, the overall methodological quality of the included studies was considered sufficient for quantitative synthesis. The updated RoB 2 assessment was conducted independently for all included studies according to the Cochrane RoB 2 framework. Detailed risk of bias assessment results are presented in Figure 2 and Supplementary Table 3.

### Effects of exercise interventions on fall-related injuries

Pooled analyses using random-effects models demonstrated that exercise interventions significantly reduced fall-related injuries among community-dwelling older adults across multiple injury outcomes.

**Figure 3:**
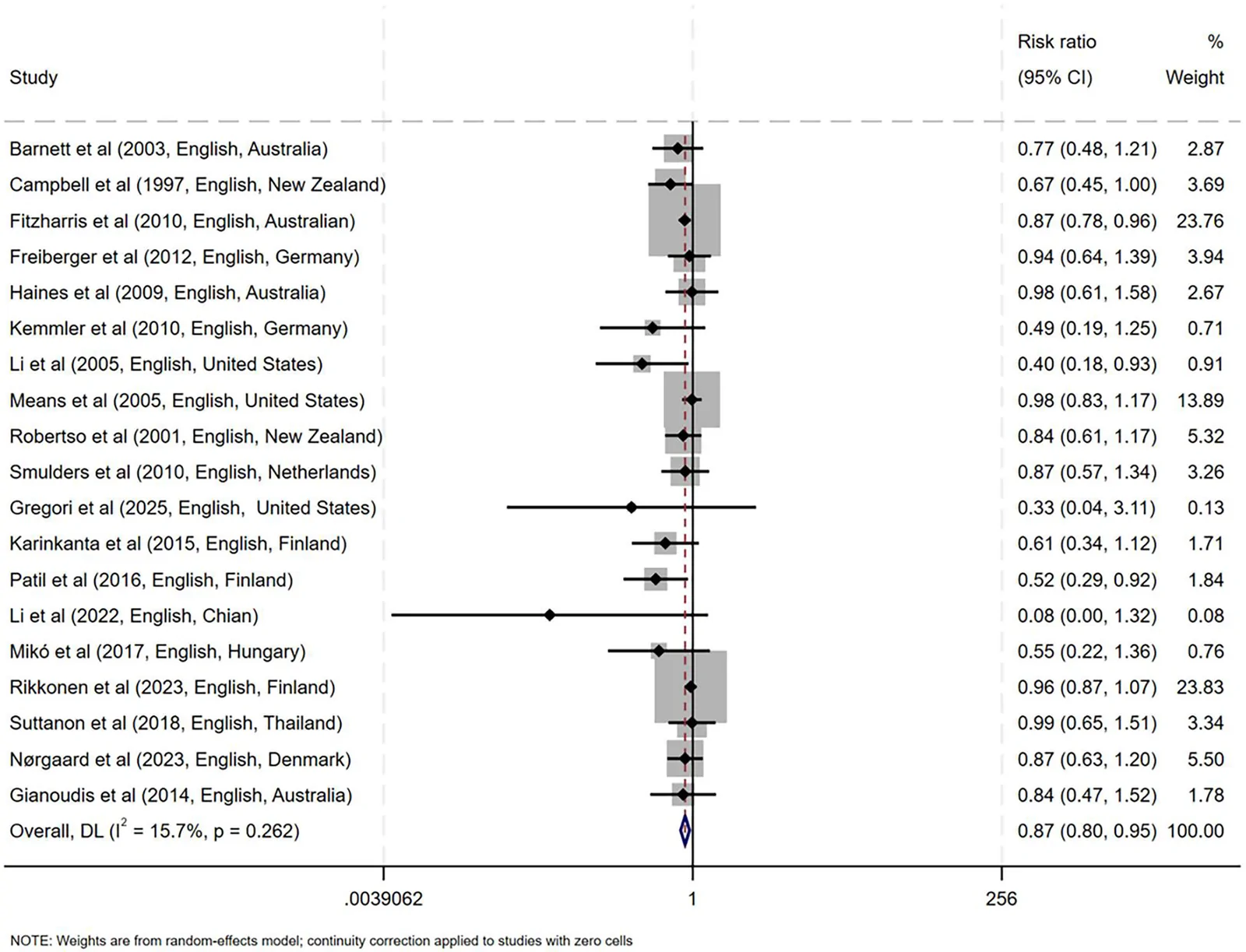
Forest plot of the effects of exercise interventions on all fall-related injuries in community-dwelling older adults.

Exercise interventions were associated with a reduced risk of all injurious falls (19 studies involving 4,690 participants and 1,956 events) (RR 0.87, 95% CI 0.80–0.95; *P* < 0.001), with low heterogeneity across studies (I^2^=15.7%). The certainty of evidence was rated as moderate according to the GRADE assessment (Figure 3 and Supplementary Table 4).

**Figure 4:**
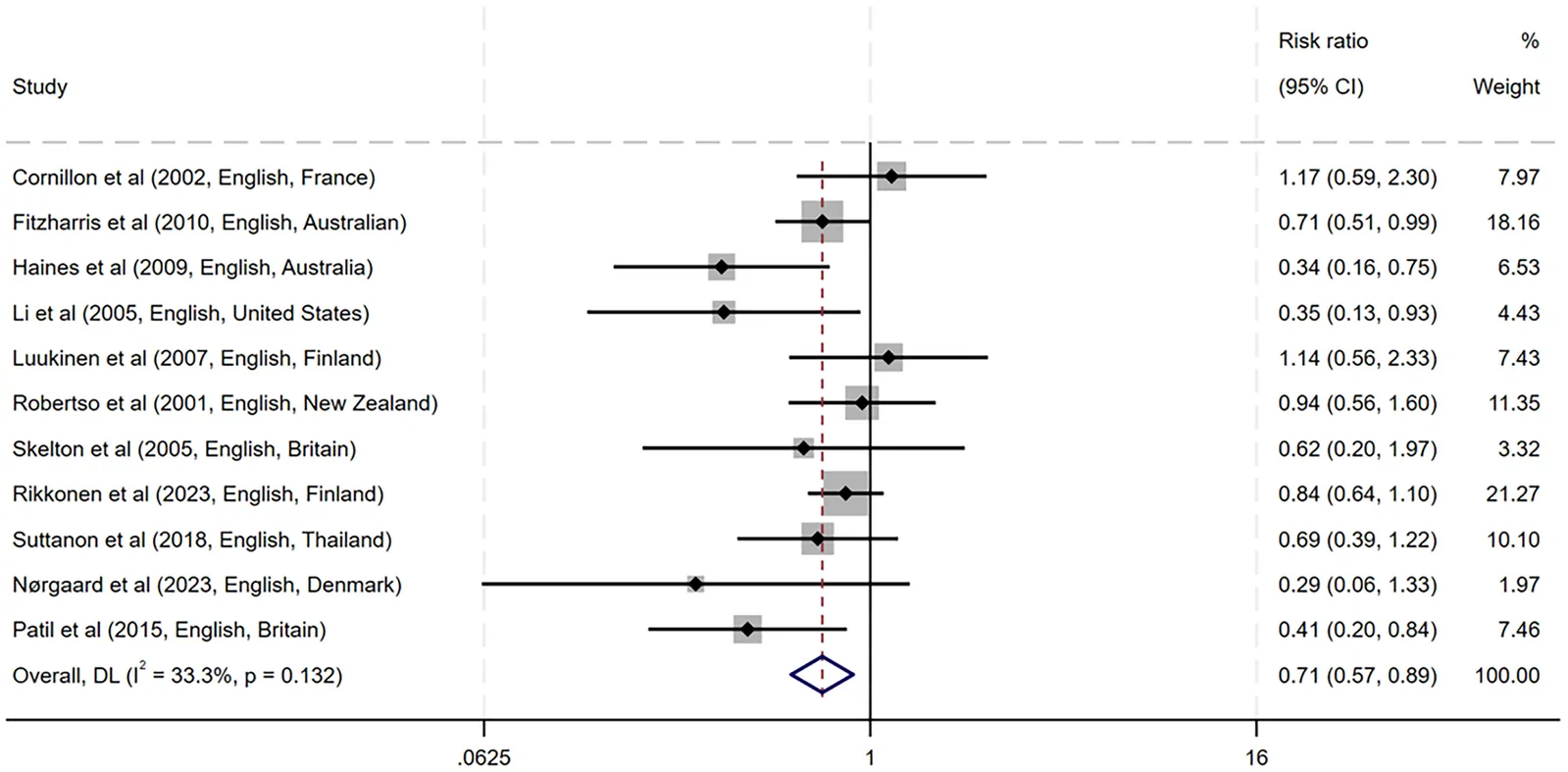
Forest plot of the effects of exercise interventions on fall-related injuries requiring medical attention in community-dwelling older adults.

Similarly, exercise interventions significantly reduced falls requiring medical care (11 studies involving 4,002 participants and 542 events) (RR 0.71, 95% CI 0.57–0.89; *P* = 0.003), although moderate heterogeneity was observed (I^2^=33.3%). The certainty of evidence was rated as low according to the GRADE assessment (Figure 4 and Supplementary Table 4).

**Figure 5:**
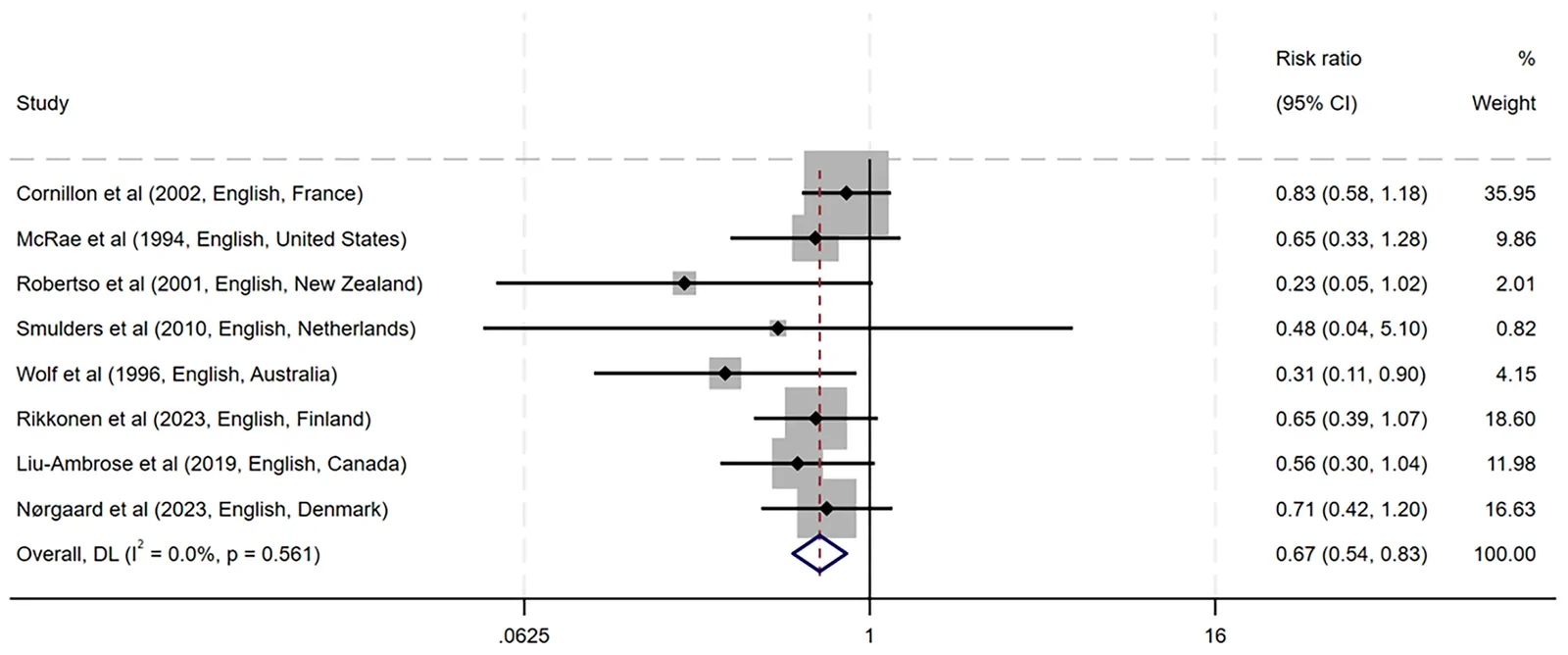
Forest plot of the effects of exercise interventions on fall-related fractures in community-dwelling older adults.

For more severe outcomes, exercise interventions demonstrated consistent protective effects. The risk of serious injurious falls was significantly reduced (8 studies involving 2,273 participants and 286 events) (RR 0.67, 95% CI 0.54–0.83; *P* < 0.001), with no substantial heterogeneity detected across studies (I^2^ = 0.0%). The certainty of evidence was rated as moderate according to the GRADE assessment (Figure 5 and Supplementary Table 4).

**Figure 6:**
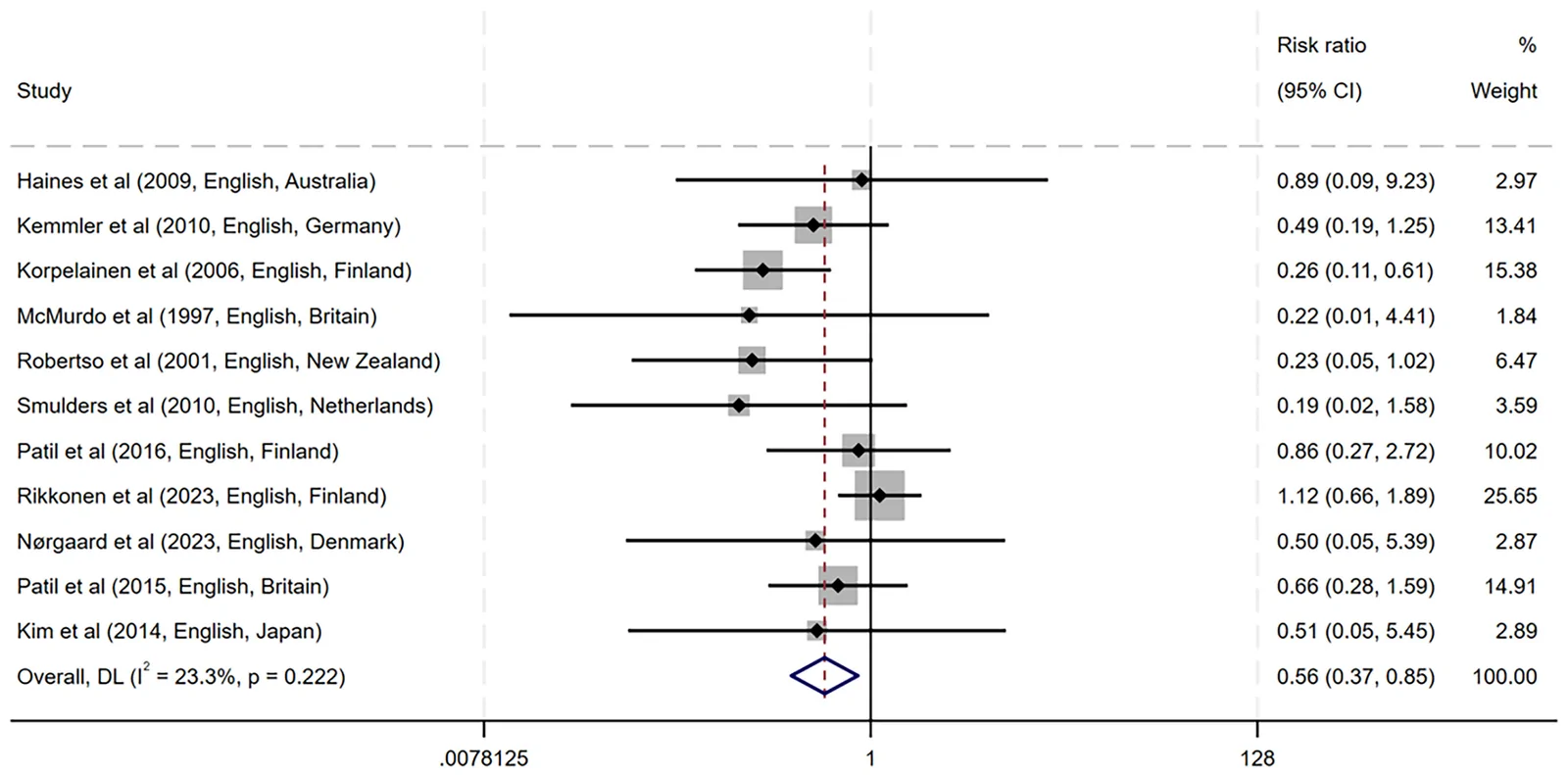
Forest plot of the effects of exercise interventions on severe fall-related injuries in community-dwelling older adults.

In addition, exercise interventions significantly decreased the risk of fall-related fractures (11 studies involving 2,630 participants and 157 events) (RR 0.56, 95% CI 0.37–0.85; *P* = 0.007), with low heterogeneity observed (I^2^=23.3%). The certainty of evidence was rated as low according to the GRADE assessment (Figure 6 and Supplementary Table 4).

Compared with the previous review published in 2013, the updated analysis incorporated additional contemporary randomized controlled trials and continued to demonstrate a significant protective effect of exercise interventions on fall-related injuries among community-dwelling older adults.

### Publication bias

**Figure 7:**
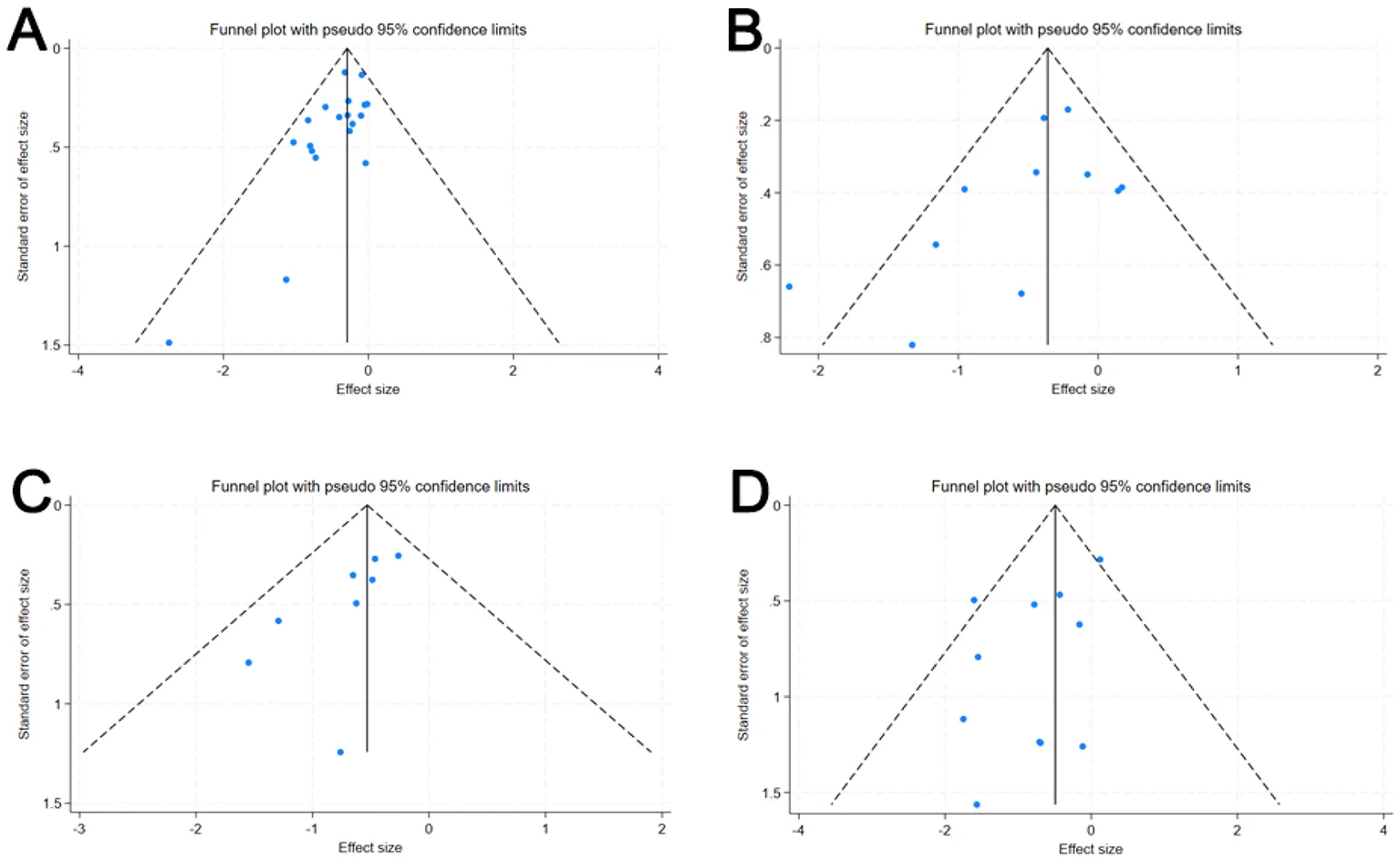
Funnel plots for publication bias assessment. **(A)** All injurious falls; **(B)** falls resulting in medical care; **(C)** falls resulting in serious injuries; and **(D)** falls resulting in fractures.

Visual examination of the funnel plots revealed no significant asymmetry between the analytical results, indicating no obvious evidence of publication bias. Funnel plots for all results are shown in Figure 7.

### Evidence certainty

The certainty of evidence for each outcome was assessed using the Grading of Recommendations Assessment, Development and Evaluation (GRADE) framework. The certainty of evidence was downgraded based on considerations including risk of bias, inconsistency, indirectness, imprecision, and publication bias.

Overall, the certainty of evidence ranged from low to moderate across the evaluated outcomes. Moderate-certainty evidence indicated that exercise interventions probably reduced all injurious falls (19 RCTs) and falls resulting in serious injuries (8 RCTs). Low-certainty evidence suggested that exercise interventions may reduce falls requiring medical care (11 RCTs) and fall-related fractures (11 RCTs). The main reasons for downgrading the certainty of evidence were concerns regarding risk of bias, imprecision, and publication bias. Detailed GRADE assessments are presented in Table 5.

**Table 5:** GRADE.

Certainty assessment						Other considerations	No. of patients		Effect		Certainty	Importance
No. of studies	Study design	Risk of bias	Inconsistency	Indirectness	Imprecision		[intervention]	[comparison]	Relative (95% CI)	Absolute (95% CI)		
All injurious falls												
19	Randomized trials	Serious^a^	Not serious	Not serious	Not serious	None	920/2,344 (39.2%)	1,036/2,346 (44.2%)	**RR 0.87** (0.80 to 0.95)	**6 fewer per 100** (from 9 fewer to 2 fewer)	⊕⊕⊕◯ Moderate^a^	IMPORTANT
Falls resulting in medical care												
11	Randomized trials	Serious^a^	serious^b^	Not serious	Not serious	None	221/1,978 (11.2%)	321/2,024 (15.9%)	**RR 0.71** (0.57 to 0.89)	**6 fewer per 100** (from 8 fewer to 2 fewer)	⊕⊕◯◯ Low^a,b^	IMPORTANT
Falls resulting in serious injuries												
8	Randomized trials	Serious^a^	Not serious	Not serious	Not serious	None	111/1,123 (9.9%)	175/1,150 (15.2%)	**RR 0.67** (0.54 to 0.83)	**6 fewer per 100** (from 7 fewer to 3 fewer)	⊕⊕⊕◯ Moderate^a^	IMPORTANT
Falls resulting in fractures												
11	Randomized trials	Serious^a^	Serious^b^	Not serious	Not serious	None	59/1,308 (4.5%)	98/1,322 (7.4%)	**RR 0.56** (0.37 to 0.85)	**4 fewer per 100** (from 5 fewer to 1 fewer)	⊕⊕◯◯ Low^a,b^	IMPORTANT

CI, confidence interval; RR, risk rati; a, Some concerns or high risk of bias in included studies; b, The study results show inconsistencies.

## Discussion

This updated systematic review and meta-analysis assessed the impact of exercise intervention on fall-related injuries in older adults in the community. Based on evidence from 29 randomized controlled trials involving 6,367 participants, our results indicate that exercise intervention significantly reduced the incidence of various fall-related injuries(7, 14), including all types of falls, falls requiring medical attention, severe falls, and fall-related fractures(14). These findings suggest that exercise interventions may be beneficial for reducing the risk of fall-related injuries among community-dwelling older adults (10).

Compared to the previous systematic review published in 2013 (14), this study included more randomized controlled trials published over the past decade and adopted updated methodological standards, including the PRISMA 2020 reporting guidelines, the Cochrane RoB 2 tool, and the GRADE framework (15–17). Despite differences in study characteristics and intervention protocols among the newly included trials, the overall protective effect of exercise intervention remained consistent. Importantly, our updated analysis continues to show significant reductions in important injury outcomes, particularly severe injuries and fractures closely associated with hospitalization, disability, loss of independent living ability, admission to aged care facilities, and death in older adults (24, 25).

Although exercise interventions are beneficial for all injury outcomes, the pooled effect estimates observed in this review are generally lower than those reported in earlier studies. This attenuation may be due to several factors. First, recent trials may better reflect real-world clinical and community settings, with broader inclusion criteria and greater population heterogeneity (26). Second, significant differences exist in exercise intensity, adherence, intervention duration, and supervision levels in contemporary studies, which may affect intervention efficacy (27, 28). Third, larger, more methodologically rigorous trials published in recent years may reduce the likelihood of effect overestimation common in smaller studies (29). Nevertheless, despite the slight decrease in pooled effect sizes, exercise interventions still demonstrate statistically significant reductions in fall-related injuries.

Importantly, preventing fall-related injuries may be more clinically significant than simply reducing fall frequency (30). While many previous studies and meta-analyses have focused primarily on fall incidence, fall injuries are the most directly related consequences for older adults in terms of emergency room visits, hospitalizations, long-term care admissions, healthcare expenditures, functional decline, and mortality (31). In this context, the observed reductions in medical-related injuries, serious injuries, and fractures highlight the broader clinical and public health significance of exercise interventions (32). Further research is needed to determine whether these benefits translate into reductions in healthcare utilization and broader economic impacts in community-based aging programs (33).

Notably, exercise interventions have shown particularly significant effects in reducing severe fall injuries and fall-related fractures (34). The lack of significant heterogeneity in the severe injury analysis provides additional support for the consistency of this finding across the included studies (35). Fractures, especially hip fractures and major osteoporotic fractures, are among the most serious consequences of falls in older adults and are often associated with long-term disability, residency in nursing homes, and excessive mortality (36, 37). Therefore, the significant reduction in fracture risk observed in this review suggests that exercise interventions may contribute to reducing the occurrence of serious fall-related outcomes (38).

The mechanisms by which exercise interventions reduce fall-related injuries are likely multifactorial. Exercise programs that include balance, strength, gait, and functional training can improve postural stability, proprioception, lower limb muscle strength, mobility, reaction time, and neuromuscular coordination (9). Consistent with recent clinical practice recommendations, multicomponent exercise programs incorporating balance and strength training may provide greater benefits for fall prevention by targeting multiple risk factors simultaneously (69). However, the included studies showed considerable variation in exercise characteristics, including intervention duration, frequency, intensity, and exercise components. Although most interventions were structured exercise programs, the optimal exercise dose and specific components associated with greater reductions in fall-related injuries remain unclear. Similarly, Kuzu et al. reported that, among older adults with chronic non-specific low back pain, adding aerobic exercise to trunk stabilization training resulted in greater improvements in functional capacity, physical performance, and fall risk compared with trunk stabilization exercises alone, suggesting the potential value of combined exercise approaches for improving functional outcomes and reducing fall risk. These physiological adaptations can reduce the likelihood of falls and the severity of injuries when they do occur. Furthermore, regular exercise helps improve bone health, slows the progression of sarcopenia, enhances cognitive-motor integration, boosts body confidence, and improves functional independence (39). These factors may collectively contribute to reducing fall-related injury risk among older adults.

Another important consideration is the increasing feasibility and scalability of exercise-based interventions in community settings. Recent trials have increasingly evaluated home-based, multi-component, and low-supervision exercise interventions. In our review, exercise interventions varied substantially in their delivery formats, ranging from supervised group-based programs to home-based approaches. This variability may partly explain differences in intervention effects across studies. However, due to the limited number of studies and heterogeneity in intervention protocols, further quantitative analyses, such as meta-regression based on exercise characteristics, were not feasible. Future research should investigate the dose-response relationship and identify the specific exercise components, duration, and intensity required to maximize reductions in fall-related injuries among community-dwelling older adults.

## Conclusion

This updated systematic review and meta-analysis demonstrates that exercise intervention significantly reduces the incidence of fall-related injuries among older adults in the community, including all types of falls, injuries requiring medical attention, severe falls, and fall-related fractures. Although the pooled effect estimate was slightly lower than that of earlier evidence-gathering analyses, the protective effect of exercise intervention remained consistent across multiple injury outcomes, suggesting a potential protective role of exercise interventions in reducing fall-related injuries among community-dwelling older adults.

These findings reinforce the role of exercise intervention as an important approach within healthy aging and fall prevention strategies. Given the heavy healthcare and social burden that fall-related injuries place on older adults, implementing accessible, scalable, and sustainable exercise programs in communities and primary care settings may contribute to supporting functional independence and improving health outcomes in older adults. However, further research incorporating economic evaluations is needed to determine the cost-effectiveness and potential impact of exercise interventions on healthcare resource utilization.

Future research should focus not only on identifying the most effective exercise characteristics but also on optimizing the long-term adherence, feasibility, and scalability of exercise interventions across different older adult populations.

## Data Availability

The original contributions presented in the study are included in the article/Supplementary material, further inquiries can be directed to the corresponding author.
