# Supplementary material for: Effects of exercise interventions on fall-related injuries in community-dwelling older adults: an updated systematic review and meta-analysis
Source: Front Public Health. 2026 Aug 26;14:1916495. doi: 10.3389/fpubh.2026.1916495 (PMC13553498; doi:10.3389/fpubh.2026.1916495)
Supplement: Supplementary file 1 [file Supplementary_file_1.docx]

Pubmed:

("Accidental Falls"[Mesh] OR fall*[Title/Abstract])

AND

("Exercise"[Mesh] OR "Motor Activity"[Mesh] OR exercise[Title/Abstract]

OR "physical activity"[Title/Abstract]

OR training[Title/Abstract]

OR "tai chi"[Title/Abstract])

AND

("Wounds and Injuries"[Mesh]

OR injur*[Title/Abstract]

OR fracture*[Title/Abstract])

AND

("Aged"[Mesh]

OR elderly[Title/Abstract]

OR "older adults"[Title/Abstract]

OR "community dwelling"[Title/Abstract])

AND

(randomized controlled trial[Publication Type]

OR randomized[Title/Abstract]

OR randomly[Title/Abstract]

OR trial[Title/Abstract])

NOT

(meta-analysis[Publication Type]

OR systematic review[Publication Type]

OR review[Publication Type])

NOT

(Alzheimer*[Title/Abstract]

OR Parkinson*[Title/Abstract]

OR dementia[Title/Abstract]

OR "nursing home"[Title/Abstract]

OR "care home"[Title/Abstract]

OR athletes[Title/Abstract])

Embase:

1. exp accidental fall/

2. (fall* OR accidental fall*).ti,ab,kw.

3. 1 OR 2

4. exp exercise/

5. exp motor activity/

6. (exercise OR "physical activity" OR training OR "tai chi" OR taichi).ti,ab,kw.

7. 4 OR 5 OR 6

8. exp wound/

9. exp injury/

10. (injur* OR fracture* OR "fall-related injur*" OR "fall-related fracture*").ti,ab,kw.

11. 8 OR 9 OR 10

12. exp aged/

13. (elderly OR "older adult*" OR "older people" OR "community dwelling" OR "community-dwelling").ti,ab,kw.

14. 12 OR 13

15. randomized controlled trial/

16. random*.ti,ab,kw.

17. trial.ti,ab,kw.

18. 15 OR 16 OR 17

19. 3 AND 7 AND 11 AND 14 AND 18

20. exp meta analysis/

21. exp systematic review/

22. review.pt.

23. 20 OR 21 OR 22

24. (Alzheimer* OR Parkinson* OR dementia OR "nursing home" OR "care home" OR athletes).ti,ab,kw.

25. 19 NOT 23

26. 25 NOT 24

Cochrane Library:

#1 MeSH descriptor: [Accidental Falls] explode all trees

#2 fall*:ti,ab,kw

#3 #1 OR #2

#4 MeSH descriptor: [Exercise] explode all trees

#5 MeSH descriptor: [Motor Activity] explode all trees

#6 exercise:ti,ab,kw

#7 "physical activity":ti,ab,kw

#8 training:ti,ab,kw

#9 "tai chi":ti,ab,kw

#10 #4 OR #5 OR #6 OR #7 OR #8 OR #9

#11 MeSH descriptor: [Wounds and Injuries] explode all trees

#12 injur*:ti,ab,kw

#13 fracture*:ti,ab,kw

#14 #11 OR #12 OR #13

#15 MeSH descriptor: [Aged] explode all trees

#16 elderly:ti,ab,kw

#17 "older adults":ti,ab,kw

#18 "community dwelling":ti,ab,kw

#19 #15 OR #16 OR #17 OR #18

#20 randomized controlled trial:ti,ab,kw

#21 random*:ti,ab,kw

#22 trial:ti,ab,kw

#23 #20 OR #21 OR #22

#24 #3 AND #10 AND #14 AND #19 AND #23

#25 meta-analysis:ti,ab,kw

#26 "systematic review":ti,ab,kw

#27 review:ti,ab,kw

#28 Alzheimer*:ti,ab,kw

#29 Parkinson*:ti,ab,kw

#30 dementia:ti,ab,kw

#31 "nursing home":ti,ab,kw

#32 "care home":ti,ab,kw

#33 athletes:ti,ab,kw

#34 #24 NOT (#25 OR #26 OR #27 OR #28 OR #29 OR #30 OR #31 OR #32 OR #33)

CINAHL:

S1

(MH "Accidental Falls+"

OR TI fall*

OR AB fall*)

AND

S2

(MH "Exercise+"

OR MH "Motor Activity+"

OR TI exercise

OR AB exercise

OR TI "physical activity"

OR AB "physical activity"

OR TI training

OR AB training

OR TI "tai chi"

OR AB "tai chi")

AND

S3

(MH "Wounds and Injuries+"

OR TI injur*

OR AB injur*

OR TI fracture*

OR AB fracture*)

AND

S4

(MH "Aged+"

OR TI elderly

OR AB elderly

OR TI "older adults"

OR AB "older adults"

OR TI "community dwelling"

OR AB "community dwelling")

AND

S5

(PT "Randomized Controlled Trial"

OR TI random*

OR AB random*

OR TI trial

OR AB trial)

NOT

S6

(TI meta-analysis

OR AB meta-analysis

OR TI "systematic review"

OR AB "systematic review"

OR TI review

OR AB review)

NOT

S7

(TI Alzheimer*

OR AB Alzheimer*

OR TI Parkinson*

OR AB Parkinson*

OR TI dementia

OR AB dementia

OR TI "nursing home"

OR AB "nursing home"

OR TI "care home"

OR AB "care home"

OR TI athletes

OR AB athletes)
