# Supplementary material for: Effects of exercise interventions on fall-related injuries in community-dwelling older adults: an updated systematic review and meta-analysis
Source: Front Public Health. 2026 Aug 26;14:1916495. doi: 10.3389/fpubh.2026.1916495 (PMC13553498; doi:10.3389/fpubh.2026.1916495)
Supplement: Supplementary file 2 [file Supplementary_file_2.docx]

**Supplementary Table 2 The PICOs details**

| **Participant** | **Intervention** | **Control** | **Outcomes** | **Studies** |
| --- | --- | --- | --- | --- |
| Studies were eligible if they included community-dwelling older adults with a mean age of 60 years or older. Studies involving participants living in nursing homes, residential care facilities, or long-term care institutions were excluded. Studies primarily targeting participants with specific neurological disorders, including Parkinson’s disease, Alzheimer’s disease, or dementia, were also excluded. | Eligible interventions included structured exercise-based programs designed to improve physical function and/or reduce falls. Interventions included balance training, resistance exercise, gait training, functional exercise, Tai Chi, multicomponent exercise, home-based exercise, supervised exercise, and group-based physical activity programs. | Usual care, health education, wait-list control, no intervention, or other non-exercise control conditions. | Studies were required to report at least one fall-related injury outcome. Outcomes were categorized as follows: All injurious falls, Falls requiring medical attention, Serious injurious falls, Fall-related fractures | Randomized controlled trials (RCTs) published in English. |
