# Supplementary material for: Effects of exercise interventions on fall-related injuries in community-dwelling older adults: an updated systematic review and meta-analysis
Source: Front Public Health. 2026 Aug 26;14:1916495. doi: 10.3389/fpubh.2026.1916495 (PMC13553498; doi:10.3389/fpubh.2026.1916495)
Supplement: Supplementary file 3 [file Supplementary_file_3.docx]

**Supplementary Table 3 Risk of bias**

| Study ID | Randomization process | Deviations from intended interventions | Missing outcome data | Measurement of the outcome | Selection of the reported result | Overall Bias |
| --- | --- | --- | --- | --- | --- | --- |
| Barnett et al (2003, English, Australia) | Some concerns | Low | Some concerns | Low | Low | Some concerns |
| Campbell et al (1997, English, New Zealand) | Low | Low | Some concerns | Low | Low | Some concerns |
| Cornillon et al (2002, English, France) | Low | Some concerns | Some concerns | Low | Low | Some concerns |
| Fitzharris et al (2010, English, Australian) | Low | Low | Low | Low | Low | Low |
| Freiberger et al (2012, English, Germany) | Low | Low | Some concerns | Low | Low | Some concerns |
| Haines et al (2009, English, Australia) | Low | Low | Low | Low | Low | Low |
| Kemmler et al (2010, English, Germany) | Low | Low | Some concerns | Low | Low | Some concerns |
| Korpelainen et al (2006, English, Finland) | Low | Low | Some concerns | Low | Low | Some concerns |
| Li et al (2005, English, United States) | Low | Some concerns | Some concerns | Low | Low | Some concerns |
| Luukinen et al (2007, English, Finland) | Low | Some concerns | Some concerns | Low | Low | Some concerns |
| McRae et al (1994, English, United States) | Some concerns | Some concerns | Low | Some concerns | Some concerns | Some concerns |
| McMurdo et al (1997, English, Britain) | Some concerns | Some concerns | Low | Some concerns | Some concerns | Some concerns |
| Means et al (2005, English, United States) | Low | Some concerns | Low | Low | Low | Some concerns |
| Robertso et al (2001, English, New Zealand) | Low | Low | Some concerns | Low | Low | Some concerns |
| Skelton et al (2005, English, Britain) | Some concerns | Some concerns | Low | Low | Low | Some concerns |
| Smulders et al (2010, English, Netherlands) | Some concerns | Some concerns | Some concerns | Low | Low | Some concerns |
| Wolf et al (1996, English, Australia) | Low | Some concerns | Low | Some concerns | Low | Some concerns |
| Gregori et al (2025, English, United States) | Low | Low | Some concerns | Low | Low | Some concerns |
| Karinkanta et al (2015, English, Finland) | Low | Some concerns | Some concerns | Low | Low | Some concerns |
| Patil et al (2016, English, Finland) | Low | Low | Low | Low | Low | Low |
| Li et al (2022, English, Chian) | Low | Some concerns | Low | Low | Low | Some concerns |
| Mikó et al (2017, English, Hungary) | Low | Some concerns | Some concerns | Low | Low | Some concerns |
| Rikkonen et al (2023, English, Finland) | Low | Some concerns | Low | Low | Low | Some concerns |
| Suttanon et al (2018, English, Thailand) | Low | Some concerns | Low | Low | Low | Some concerns |
| Liu-Ambrose et al (2019, English, Canada) | Low | Some concerns | Low | Low | Low | Some concerns |
| Nørgaard et al (2023, English, Denmark) | Low | Low | Low | Low | Low | Low |
| Patil et al (2015, English, Britain) | Low | Some concerns | Some concerns | Low | Low | Some concerns |
| Gianoudis et al (2014, English, Australia) | Low | Low | Some concerns | Low | Low | Some concerns |
| Kim et al (2014, English, Japan) | Low | Low | Low | Low | Low | Low |
