# Supplementary material for: Effects of exercise interventions on fall-related injuries in community-dwelling older adults: an updated systematic review and meta-analysis
Source: Front Public Health. 2026 Aug 26;14:1916495. doi: 10.3389/fpubh.2026.1916495 (PMC13553498; doi:10.3389/fpubh.2026.1916495)
Supplement: Supplementary file 4 [file Supplementary_file_4.docx]

| Outcomes | Rate Ratio  IV Random 95% CI, I^2^ | P value |
| --- | --- | --- |
| all injurious falls | 0.87 [0.8 to 0.95], I² = 15.7% | P＜0.001** |
| falls resulting in medical care | 0.71 [0.57 to 0.89], I² = 33.3% | P=0.003** |
| falls resulting in serious inju | 0.67 [0.54 to 0.83], I² =0.0% | P＜0.002** |
| for falls resulting in fracture | 0.56 [0.37 to 0.85], I² = 23.3% | P=0.007** |

**Supplementary Table 4 Results of research on four types of fall injuries**

**Note**: Difference *: p ＜ 0.05；**: p ＜ 0.01
